# Entanglement Length Scale Separates Threading from Branching of Unknotted and Non-concatenated Ring Polymers in Melts

**DOI:** 10.1021/acs.macromol.2c01264

**Published:** 2022-11-28

**Authors:** Mattia Alberto Ubertini, Jan Smrek, Angelo Rosa

**Affiliations:** † Scuola Internazionale Superiore di Studi Avanzati (SISSA), Via Bonomea 265, 34136Trieste, Italy; ‡ Faculty of Physics, University of Vienna, Boltzmanngasse 5, A-1090Vienna, Austria

## Abstract

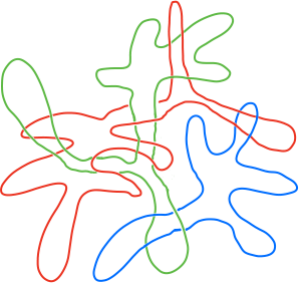

Current theories on the conformation and dynamics of unknotted and non-concatenated ring polymers in melt conditions describe each ring as a tree-like double-folded object. While evidence from simulations supports this picture on a single ring level, other works show pairs of rings also thread each other, a feature overlooked in the tree theories. Here we reconcile this dichotomy using Monte Carlo simulations of the ring melts with different bending rigidities. We find that rings are double-folded (more strongly for stiffer rings) *on and above* the entanglement length scale, while the threadings are localized *on smaller scales*. The different theories disagree on the details of the tree structure, i.e., the fractal dimension of the backbone of the tree. In the stiffer melts we find an indication of a self-avoiding scaling of the backbone, while more flexible chains do not exhibit such a regime. Moreover, the theories commonly neglect threadings and assign different importance to the impact of the progressive constraint release (tube dilation) on single ring relaxation due to the motion of other rings. Despite that each threading creates only a small opening in the double-folded structure, the threading loops can be numerous and their length can exceed substantially the entanglement scale. We link the threading constraints to the divergence of the relaxation time of a ring, if the tube dilation is hindered by pinning a fraction of other rings in space. Current theories do not predict such divergence and predict faster than measured diffusion of rings, pointing at the relevance of the threading constraints in unpinned systems as well. Revision of the theories with explicit threading constraints might elucidate the validity of the conjectured existence of topological glass.

## 1 Introduction

Topological constraints emerging from the mutual uncrossability between distinct chain segments dominate the viscoelastic behavior of polymer systems in high-density (melt) conditions.^1−3^ In this context, of notable interest are those situations where polymers are prepared in a well-defined topological state which remains quenched as polymers diffuse and flow. The simplest example in this respect, and the central topic of the present work, is the case of melts of *unknotted* and *non-concatenated* ring polymers. People have been now studying this particular class of polymer solutions for several decades, from the theoretical^4−27^ as well as the experimental^28−33^ point of view. Researchers have shown that there exist intriguing conceptual connections between melts of ring polymers and, for instance, chromosomes^34,35^ and a polymer glass based on the nonlinear topology of the chains.^36−39^ Yet, because of the particular nature of the problem, many fundamental aspects concerning the physics of ring polymer melts remain poorly understood. At present in fact, physical theories taking exactly into account the constraint by means of suitably topological invariants^40−44^ remain mathematically hard, if not completely intractable, problems, and their applicability to the dense, many-chain systems is limited. Therefore, the various physical pictures that have been proposed so far^4,5,7,15,19^ introduce suitable approximations to deal with the constraint which make the problem more affordable but, inevitably, they require a supplement of validation, either from experiments or from numerical simulations.

**Figure 1:**
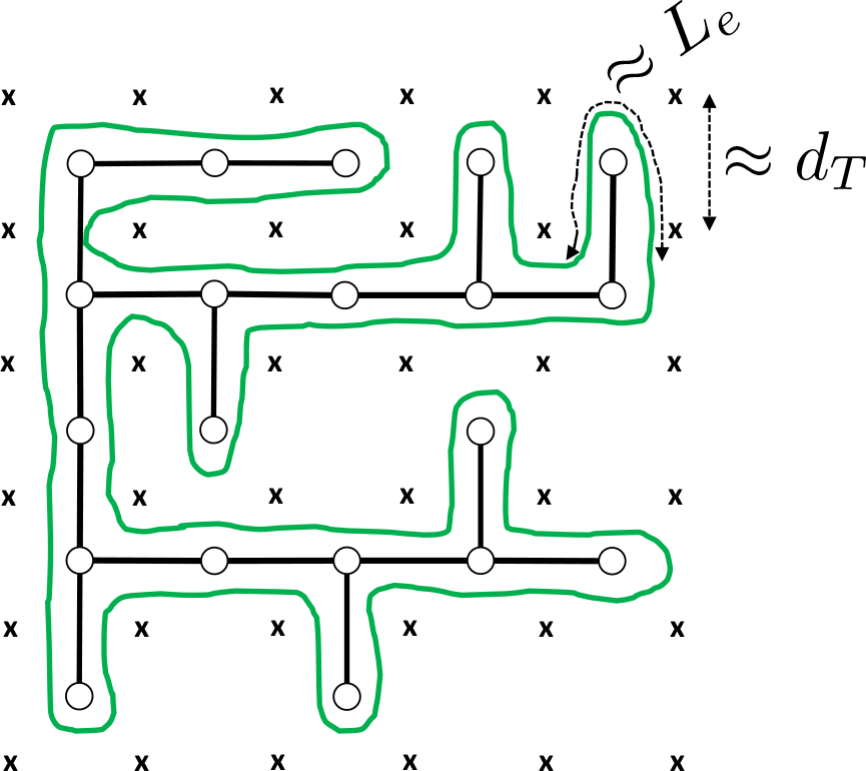
Schematic picture of a single ring polymer conformation (green line) in a melt which double-folds around its tree-like backbone. The crosses (×) represent the lattice of topological obstacles created by the surrounding rings in the melt. The lattice spacing is on the order of the tube diameter *d*_*T*_, and the polymer contour length inside each unit cell is . Random displacements of the loops protruding from the mean path on the backbone of the branched structure (thick black line) change the ring’s shape and dominate melt dynamics at long time scales.^7,16^

In a series of landmark papers^4,5,7^ topological constraints have been approximated, in a mean-field fashion, as a lattice of infinitely thin impenetrable obstacles (see Figure 1); therefore, in avoiding concatenation, rings should protrude through them by *double-folding* on *branched, tree-like* conformations. As a consequence,^13,18,24^ rings form compact shapes whose mean linear size or gyration radius, ⟨*R*_*g*_⟩, scales with the total contour length of the chain, *L*, as
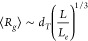
1for *L* larger than the characteristic and material-dependent^45^ contour length scale, *L*_*e*_, known as the *entanglement length*, and with *d*_*T*_, the *tube diameter* of the melt, equivalent to the average mesh distance of the array of topological obstacles.^1−3^ Many nontrivial predictions of the lattice-tree model—especially single-chain ones as, for instance, the scaling behavior of the rings (eq 1) or their monomer diffusion in the melt—appear in good agreement with brute-force dynamic simulations.^6,8−10,16,20,24^ Notably, the model has also helped cast a multiscale algorithm^14^ for generating ring melt conformations at negligible computational cost.

While seemingly accurate, the double-folding/branching model (Figure 1) dismisses^22,23^ the possibility of ring–ring interpenetrations (or, *threadings*) which instead—and without violating the non-concatenation constraint!—have physical consequences which become particularly evident when melts are driven out-of-equilibrium, such as in an elongation flow^26^ or for an induced asymmetry in the local monomer mobilities.^39^ Moreover, from a broader perspective it remains unclear if the entanglement scales *L*_*e*_ and *d*_*T*_ (eq 1) are the only relevant ones for melts of rings or if, and up to what extent, they are influenced by the local bending rigidity or Kuhn length^3^_*K*_ of the chain. In fact, the value of *L*_*e*_ is measured from a linear melt (e.g., by primitive path analysis^46^) and the length scale is only *assumed* to be applicable for rings (with the same polymer model) as well, although no *direct* method to find the value explicitly from the rings is known. Some recent comparisons of crazing in linear and ring glasses suggest^47,48^ that *L*_*e*_(rings)/*L*_*e*_(linear) ≈ 4, but its role in equilibrium ring melts, as well as its connection to _*K*_, has not been thoroughly investigated. All these aspects (double-folding/branching, threadings, entanglement scales) are clearly all related to each other and, yet, how they mutually influence each other remains poorly understood.

In order to address these questions, in this work we perform a systematic investigation of the static and dynamic properties of melts of unknotted and non-concatenated ring polymer conformations obtained in large-scale computer simulations. To this purpose, we present first a modified version of the kinetic Monte Carlo algorithm described in refs (49) and (50) with the purpose of achieving higher values of the polymer Kuhn length _*K*_: in doing so, we built on the numerically efficient strategy originally devised in ref (8), which allows us to increase substantially the overlap between polymer chains and to reach feasibly the asymptotic regime eq 1, with relatively moderate chain sizes and, hence, computing times. Then, by employing established tools like the polymer mean gyration radius and shape,^51^ contour length correlations^8^ and spatial contacts,^52^ spanned surface,^23^ and minimal surface,^17,53^ alongside the motion of the melt for unperturbed conditions^54^ or after partial pinning^37^ of a given fraction of the polymer population, we find out that rings are systematically threading each others via the formation of locally double-folded structures on length scales below the tube diameter *d*_*T*_. Additionally, we show that the threading loops of contour length  (which we name *shallow threadings*) influence significantly many, nonuniversal, properties of the rings.

The paper is structured as follows. In Section 2, we present the notation employed in this work (Section 2.1) and summarize the main predictions for the structure (Section 2.2) and the dynamics (Section 2.3) of melts of rings according to various models with the emphasis on their lattice-tree features. In Section 3, we present and discuss our lattice polymer model and derive the relevant length and time scales of the polymer melts. In Section 4 we present the main results, for the structure (Section 4.1) and the dynamics (Section 4.2) of the rings. In these sections and in the final Section 5 we present and discuss how the tree-like models can be reconciled with the threading features. We also indicate measurements that can help to discriminate between the different models and discuss the common deficiency of all of them. Additional tables and figures are included in the Supporting Information (SI).

## 2 Ring Polymers in Melt: Theoretical Background

### 2.1 Entanglement Length and Time Units: Definitions and Notation

Single polymers in melt are made of a linear sequence of monomer units with mean bond length = ⟨*b*⟩. The total number of monomers of each polymer chain is = *N* and the polymer contour length is *L* = *N*⟨*b*⟩, while we denote by  (≤*L*) the contour length of a polymer subchain made of *n* ≡ /⟨*b*⟩ ≤ *N* monomers.

Topological constraints (entanglements) affect polymer conformations in the melt when the total contour length of each chain, *L*, exceeds the characteristic entanglement length scale *L*_*e*_. In general *L*_*e*_ is a nontrivial function of the chain bending stiffness or the Kuhn length, _*K*_, and the Kuhn segment density, ρ_*K*_, of the melt (see eq 19 and ref (46)). Then, the mean gyration radius of the polymer chain of contour length *L* = *L*_*e*_ and Kuhn length _*K*_,
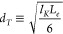
2is of the order of the cross-sectional diameter of the *tube-like* region^1−3^ where the polymer is confined due to the presence of topological constraints. Unless otherwise said, we will express chain contour lengths *L* in units of the entanglement length *L*_*e*_ and, to this purpose, we introduce the compact notations^14,23^
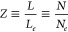
3
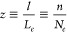
4where *N*_*e*_ corresponds to the number of monomers *L*_*e*_/⟨*b*⟩ of an entanglement length and *Z* and *z* are the number of entanglements of the polymer chain and the polymer subchain of contour lengths *L* and , respectively.^55^ Accordingly, variables such as the polymer mean-gyration radius ⟨*R*_*g*_⟩ or the mean-magnetic radius ⟨*R*_*m*_⟩ (see definitions eqs 20 and 21, respectively) are expressed in units of the tube diameter *d*_*T*_ (eq 2).

With respect to polymer dynamics, entanglements affect the motion of the chains on time scales τ larger than a characteristic time τ_*e*_, known as the *entanglement time*. τ_*e*_ is defined as in ref (54); namely it corresponds to the time scale where the monomer mean-square displacement *g*_1_(τ = τ_*e*_) (eq 8) is  (eq 2). From now on, we adopt τ_*e*_ as our main unit of time.

In Section 3.4, we present a detailed derivation of these quantities for the polymer melts considered in this work.

### 2.2 Ring Structure

According to the lattice-tree picture,^4,5,7,13,14,16^ ring conformations in the melt are the result of the balance between compression to avoid linking with other rings and swelling to avoid self-knotting. At the same time, this size competition can be viewed as a balance between double-folding (which minimizes threadings between chains) and random branching (which maximizes polymer entropy). As a consequence, a melt of rings can be mapped^14^ to an equivalent melt of randomly branching polymers or lattice trees with the same large-scale behavior (see Figure 1). In turn, this mapping can be employed to derive quantitative predictions^13,18,24,56^ for the scaling exponents describing the characteristic power-law behaviors as a function of *L*/*L*_*e*_ ≡ *Z* ≳ 1 of the following observables:

(i) The mean ring size or gyration radius (eq 1) as a function of the ring mass:
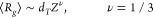
5
(ii) The mean path length on the backbone of the tree (Figure 1, thick black line) as a function of the ring mass:
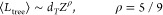
6
(iii) The mean ring size as a function of the mean path length:
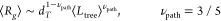
7

Equation 5 means that rings (i.e., the equivalent trees) behave like compact, space-filling objects, while eq 7 expresses the fact that linear paths follow self-avoiding walk statistics.^13^ Notice also that eq 7 follows directly from eqs 5 and 6; hence ν_path_ = ν/ρ. While the model explains and quantifies the origin of branching and correctly predicts the measured exponent ν in the asymptotic limit, there have been no attempts so far to measure the exponents ρ or ν_path_ in simulations directly (the backbone is not easily “visible” in the conformations). Moreover, the theory is based on scales above *N*_*e*_ and states explicitly^16^ that the branches might not be double-folded to the monomer scale. This means that the branches can open up to sizes about *d*_*T*_ to allow for a branch of another ring to thread through. As we will show in Section 4.1, this is indeed the case.

Another very popular and accurate model is the so-called fractal loopy globule (FLG).^19^ The model is based on the conjecture that the ring structure arises from a constant (and equal to the Kavassalis–Noolandi number^57^) overlap parameter (number of segments/loops of a given length sharing a common volume) on all scales above *L*_*e*_ in a self-similar manner. As a result, the exponent ν = 1/3 is practically postulated. As such, the ring structure, as loops on loops, is “somewhat analogous” to a randomly branched structure, where the loops of the FLG are viewed as the branches. The authors assert that the loops are not perfectly double-folded, but do not elaborate on the double-folded structure further, except that the number of loops/branches *n*(*r*) of size *r* per ring is *n*(*r*) ∼ *r*^–3^.

The difference of the FLG and the lattice-tree models, from the structural point of view, is in the branching statistics, manifested in the structure of the mean (primitive) path. [Note that here we use the terms primitive path and tree backbone interchangeably, because they have the same meaning for linear polymers: the shortest end-to-end path of the chain to which its contour can be contracted without crossing other chains. In rings, there are no ends, and therefore such equivalence is, to say the least, not clear. The tree backbone, governing the stretching and branching of the ring is a properly weighted average of all possible path lengths in the tree structure (see ref (16) for details), while the primitive path of the ring is measured in ref (19) with a method analogous, but not identical, to primitive path analysis.^46^] While in the lattice-tree model the scaling of the size of a segment of the ring with the corresponding primitive path is governed by the exponent ν_path_ on scales above *N*_*e*_ (7), in the FLG the scaling is more complicated because it takes into account “tube dilation”. The tube dilation means freeing of the constraints imposed by the other surrounding segments due to their motion, hence effectively increasing the length scale between constraints, making it time-dependent *N*_*e*_(*t*). In the FLG model, if the segment is shorter than *N*_*e*_(*t*), its primitive path is just straight; hence the size of the segment scales linearly with the length. If the segment is longer than *N*_*e*_(*t*), its size scales with the exponent ν = 1/3 equal to that of the whole ring. The analysis of the primitive paths in ref (19) reports that they do not observe the scaling of the size of the primitive path with its length with the exponent ν_path_ = 3/5. Yet their analysis focuses on a rather flexible system only, and the reported dependence broadly and smoothly crosses over from the exponent 1 to 1/3 (hence visiting all the intermediate exponents). As we show below in Section 4.1, we find an indication of the exponent ν_path_ = 3/5 in the stiff melts.

### 2.3 Ring Dynamics

As originally pointed out by Obukhov et al.,^7^ the branched structure induced by the topological obstacles (Figure 1) has direct implications for ring dynamics as well. Here, we limit ourselves to a schematic description of the physics of the process and to recapitulate the main results, without entering into a detailed derivation which, being far from trivial, the interested reader can find explained in detail in refs (7), (16), and (27).

In order to quantify chain dynamics, we introduce^54^ the monomer mean-square displacement
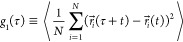
8and the mean-square displacement of the ring center of mass
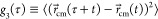
9as a function of time τ. Then, three regimes can be identified. For time scales τ ≲ τ_*e*_ and length scales ≲ *d*_*T*_, monomer motion is not affected by entanglements, and we expect the characteristic Rouse-like^3^ behavior, *g*_1_(τ) ∼ τ^1/2^ and *g*_3_(τ) ∼ τ. On intermediate time scales τ ≳ τ_*e*_, the motion of the ring is dominated by mass transport along the mean path on the tree-like backbone (Figure 1). Finally, for large time scales τ ≳ τ_*r*_ where τ_*r*_ ≈ τ_*e*_*Z*^2+ρ^ is the global relaxation time of the chain,^16^ the whole chain is simply diffusing, i*.*e*.*, *g*_1_(τ) ∼ *g*_3_(τ) ∼ τ. The complete expressions for *g*_1_ and *g*_3_, up to numerical prefactors but including the coefficients for smooth crossovers,^16^ are given by
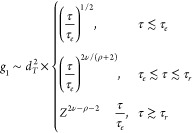
10and
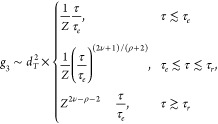
11where the exponents ν and ρ appearing here are the same ones introduced in the scaling behaviors of the static quantities; see Section 2.2.

Finally, we introduce the monomer mean-square displacement in the frame of the chain center of mass:
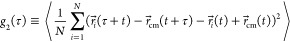
12

It is not difficult to see that *g*_2_(τ) ≃ *g*_1_(τ) – *g*_3_(τ) and that  where  is the chain mean-square gyration radius (see definition, eq 20). We adopt the appearance of a plateau in the large-time behavior of *g*_2_ as the signature that our chains have attained complete structural relaxation (see Figure S1 in the SI).

The FLG model^19^ takes into account the tube dilation, as mentioned in Section 2.1, but otherwise uses self-similar dynamics characteristics for other branched ring conformations as well. Therefore, as shown in ref (19), the predictions for the *g*_1_ of both models, the lattice-tree dynamics and the FLG, can be concisely written as
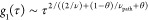
13for times τ_*e*_ ≤ τ ≤ τ_*r*_. The parameter θ governs the tube dilation: θ = 0 means no tube dilation as in the lattice-tree model, while θ = 1 means full tube dilation as in the FLG model. Note that in FLG, because of the full tube dilation the exponent ν_path_ does not impact the dynamics. The exponents are 0.26 for the lattice-tree model and about 0.29 for FLG.

Interestingly both the FLG and the annealed lattice-tree model are very close in the predictions of the scaling of the monomer mean-squared displacements and other dynamical quantities,^19^ apart from the scaling of the diffusion coefficient with *N*. This is computed as *D* ∼ *R*^2^/τ_*r*_, where the relaxation time τ_*r*_ involving the tube dilation can be written as
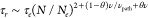
14which gives
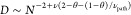
15

Although the exponent is difficult to measure accurately in simulations due to a need of very long runs, the theories underestimate the exponent obtained from the simulations^10,19^ as well as that from the experiments^33^ by 0.67 and 0.45 for the FLG and the lattice-tree model, respectively. As we show below in Section 4.2, the source of the discrepancy can be caused by the threadings.

## 3 Polymer Model and Numerical Methods

### 3.1 Melts of Rings: The Kinetic Monte Carlo Algorithm

We model classical solutions of ring polymers of variable Kuhn length _*K*_ and with excluded volume interactions by adapting the kinetic Monte Carlo (kMC) algorithm for elastic lattice polymers on the three-dimensional face-centered-cubic (fcc) lattice introduced originally in refs (49) and (50) and employed later in several studies on polymer melts.^23,58,59^ In the following we provide the main features of the model, while we refer the reader to the mentioned literature for more details.

In the model, any two consecutive monomers along the chain sit either on nearest-neighbor lattice sites or on the same lattice site (with no more than two consecutive monomers occupying the same lattice site), while nonconsecutive monomers are never allowed to occupy the same lattice site due to excluded volume. By adopting the lattice distance *a* between fcc nearest-neighbor sites as our unit distance, the bond length *b* between nearest-neighbor monomers fluctuates between *a* and 0 (the latter case corresponding to a unit of *stored length*): for an average bond length = ⟨*b*⟩, a polymer chain with *N* bonds has then a total contour length *L* = *N*⟨*b*⟩ < *Na*. Thanks to this numerical “trick”, polymers are made effectively elastic.^49^

Ring polymers in the melt are asymptotically compact (eq 1), yet reaching this regime requires the simulation of very large rings, which, in turn, may imply prohibitively long^8,9,14^ equilibration times. In order to overcome this limitation and, yet, still achieve substantial overlap between the different polymer chains for moderate chain lengths and, hence, feasible simulation times, we adopt the efficient strategy described in ref (8) and consider polymer chains which are locally stiff, namely, polymers whose Kuhn length^3^_*K*_ is significantly larger than the mean bond length ⟨*b*⟩. To this purpose, we have complemented the chain Hamiltonian by introducing the bending energy term:
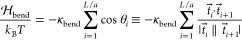
16where κ_bend_ represents the bending stiffness which determines _*K*_ (see Section 3.4), and  is the oriented bond vector between monomers *i* and *i* + 1 having spatial coordinates  and . [For ring polymers, it is implicitly assumed the periodic boundary condition along the chain *N* + 1 ≡ 1.] Importantly, since bond vectors are obviously ill-defined when two monomers form a stored length, the sum in eq 16 is restricted to the *effective* bonds of the chains. By increasing κ_bend_, the energy term eq 16 makes polymers stiffer.

Then, the dynamic evolution of the melts proceeds according to the following Metropolis–Hastings-like^60^ criterion. One monomer is picked at random and displaced toward one of the nearest lattice sites. The move is accepted based on the energy term eq 16 and if, at the same time, *both* chain connectivity and excluded volume conditions are not violated: in particular, the latter condition is enforced by imposing that the destination lattice site is either empty or, at most, occupied by one and only one of the nearest-neighbor monomers along the chain. In practice, it is useful to make the following conceptual distinction concerning how this move effectively implements classical polymer dynamics.^1−3^ The selected monomer which is displaced toward an empty or a single-occupation site (or, a Rouse-like move) may, in general, change the local chain curvature and, hence, depend on the bending energy term eq 16. Conversely, a unit of stored length traveling along the chain (or, a reptation-like move) is not affected by the curvature because eq 16 is, again, strictly restricted to the effective bonds of the chains. Overall, as explained in detail in ref (50), the stored length method ensures that the algorithm remains efficient even when it is applied to the equilibration of very dense systems. The specific values for the acceptance rates as a function of the bending rigidity κ_bend_ are summarized in Table 1.

**Table 1:** Values of Physical Parameters Characterizing the Melts of Polymers Studied in This Paper^a^

κ_bend_	acc. rate	⟨ cos θ⟩^lin^	⟨ cos θ⟩^ring^	⟨*b*⟩/*a*	*l*_K_/*a*	ρ_K_*l*_K_^3^	*L*_*e*_/*a*	*N*_*e*_	*d*_*T*_/*a*	τ_*e*_/τ_MC_(×10^4^)
0	0.069	0.187	0.171	0.731	1.440	2.679	58.738	80.379	3.755	15.0
1	0.048	0.476	0.447	0.696	2.194	5.920	20.708	29.764	2.752	5.2
2	0.036	0.670	0.635	0.669	3.393	13.620	8.759	13.088	2.226	2.8

a *a* is the unit distance of the fcc lattice, and the monomer number per unit volume is *ρa*^3^ ≃ 1.77 (Section 3.2). (i) κ_bend_, bending stiffness parameter (eq 16); (ii) acceptance rate of the kMC algorithm; (iii, iv) ⟨cos θ⟩^lin/ring^, mean value of the cosine of the angle between two consecutive bonds along the linear/ring polymer (eq 16); (v) ⟨*b*⟩, mean bond length; (vi) _*K*_, Kuhn length (eq 18); (vii) , number of Kuhn segments per Kuhn volume;^61^ (viii) *L*_*e*_, entanglement length (eq 19); (ix) *N*_*e*_ ≡ *L*_*e*_/⟨*b*⟩, number of bonds per entanglement length; (x) , diameter of the effective tube confining polymer chains in the melt; (xi) τ_*e*_, entanglement time.

### 3.2 Melts of Rings: Simulation Details

We have studied bulk properties of dense solutions of *M* closed (ring) polymer chains made of *N* monomers or bonds per ring. By construction, rings are *unknotted* and *non-concatenated*. We simulate values
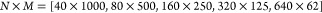
with fixed total number of monomers = 40 000 (for computational convenience, the total number of monomers for *N* = 640 is slightly less). All these systems have been studied for the bending stiffness parameters κ_bend_ = 0, 1, 2 (eq 16). In addition, we have also simulated melts with  for κ_bend_ = 1 and  for κ_bend_ = 2: once rescaled in terms of the corresponding entanglement units (see Section 3.4 and Table 1), these two setups have the same number of entanglements per chain *Z* ≈ 8 of *N* = 640 with κ_bend_ = 0.

Bulk conditions are implemented through the enforcement of periodic boundary conditions in a simulation box of total volume . Similarly to previous works,^23,50,59^ melt conditions correspond to fixing the monomer number per fcc lattice site to (i)  for *N* ≤ 320 and (ii)  for *N* = 640, respectively, since the volume occupied by the fcc lattice site is ,^62^ the monomer number per unit volume is given by (i)  for *N* ≤ 320, and (ii)  for *N* = 640. Accordingly, we fix  for all *N*’s.

Finally, our typical runs amount to a minimum of 7 × 10^6^ up to a maximum of 3 × 10^8^ kMC time units where one kMC time unit τ_MC_ is = *NM*: these runs are long enough such that the considered polymers have attained proper structural relaxation (see Figure S1 in the SI).

### 3.3 A Closer Look at the Bending Stiffness

It is worth discussing in more detail the consequences of the energy term, eq 16.

On the fcc lattice, the angle θ between consecutive chain bonds is restricted to the five values 0°, 45°, 90°, 135°, and 180°. Otherwise, for ideal polymers (i.e., in the absence of the excluded volume interaction), the angles θ_*i*_ in eq 16 are obviously not correlated to each other. This implies that the distribution function *P*_ideal_(κ_bend_; cos θ) of the variable cos θ follows the simple Boltzmann form:
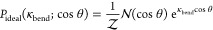
17where  represents the total number of lattice states for two successive bonds with given cos θ and  is the normalization factor.

**Figure 2:**
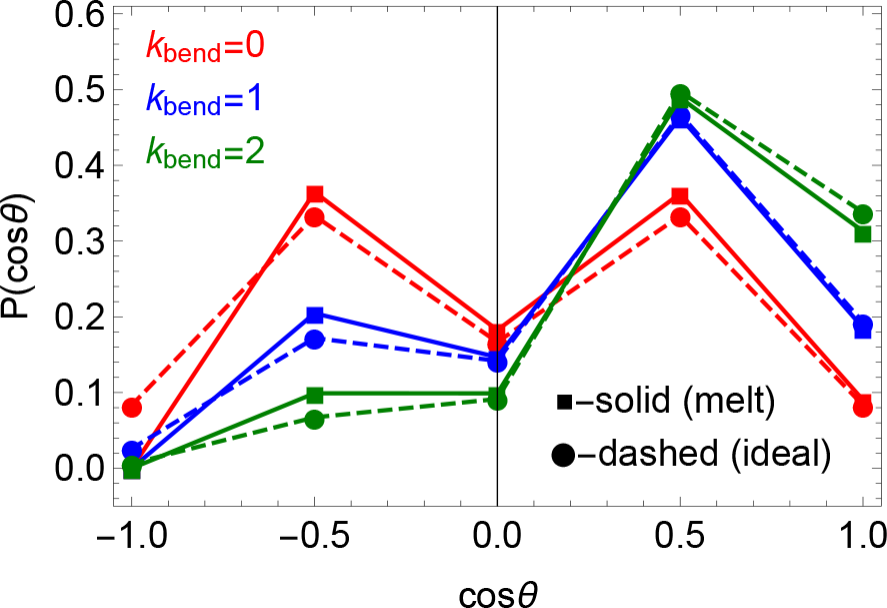
Distribution functions  (square-solid lines) of the cosine of the angle θ between consecutive bonds along the polymer chain in ring melts for different values of the bending stiffness parameter κ_bend_. The curves are compared to the results for ideal rings,  (eq 17, circle-dashed lines).

In polymer melts, excluded volume interactions induce an effective long-range coupling between bond vectors and the distribution function *P*_melt_(κ_bend_; θ) is expected to deviate from eq 17: for instance, one can immediately see that the angle = 180° (i.e., cos θ = −1) is possible for ideal polymers but strictly forbidden in the presence of excluded volume interactions. The distributions *P*_melt_(κ_bend_; θ) for the different κ_bend_’s and in comparison to *P*_ideal_(κ_bend_; θ) are given in Figure 2 (square-solid vs circle-dashed lines, respectively). Corresponding mean values ⟨cos θ⟩ for the different κ_bend_’s and for open linear chains and closed rings are summarized in Table 1: the values for the two chain architectures are very similar, with differences in the range of a few percent.

### 3.4 Polymer Length and Time Scales

We provide here a detailed derivation of the relevant length and time scales characterizing our polymer melts (they are summarized in Table 1). Since we work at fixed monomer density ρ (see Section 3.2), the values of these parameters depend only on the bending stiffness parameter κ_bend_.

#### Average Bond Length, ⟨*b*⟩

We observe that ⟨*b*⟩ is slightly decreasing with κ_bend_: arguably, this is the consequence of the progressive stiffening of the polymer fiber on the fcc lattice which privileges less kinked conformations through the reduction of the effective total contour length of the chain.

#### Kuhn Length, _*K*_

**Figure 3:**
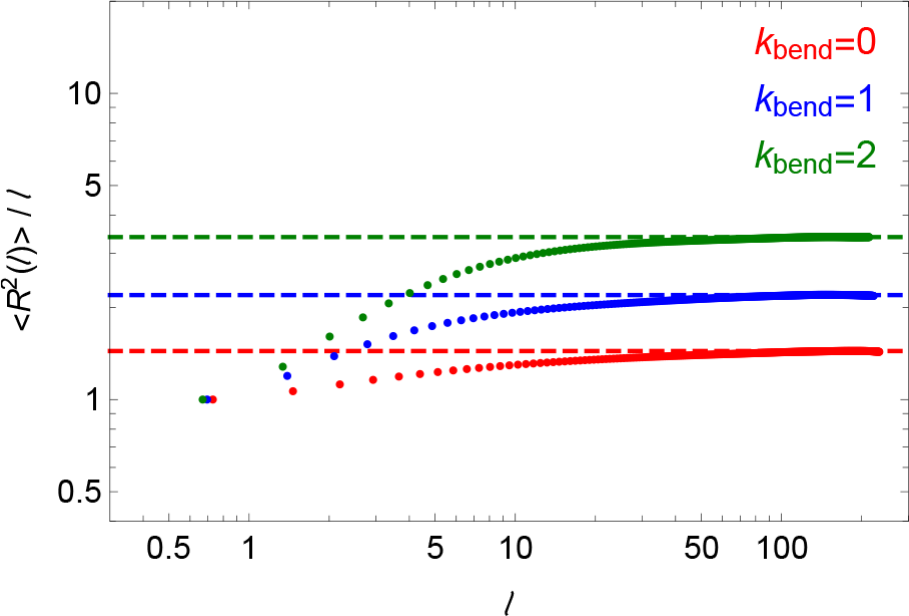
(Symbols) Ratios of the mean-square end-to-end distance, ⟨*R*^2^()⟩/, to the corresponding monomer contour length separation  for linear chains in the melt and for the different bending stiffness parameters κ_bend_ (see legend). (Dashed lines) Best fits of the plateau-like behaviors on the contour length interval  ∈ [150, 200] (see eq 18 and the text for details).

By modulating the bending constant κ_bend_, we can fine-tune the flexibility of the polymers (eq 16). The latter is quantified in terms of the Kuhn length _*K*_, namely, the unit of contour length beyond which the chain orientational order is lost.^2,3^ For linear chains, _*K*_ is defined by the relation^2,3^
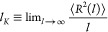
18where ⟨*R*^2^()⟩ is the mean-square end-to-end distance between any two monomers along the chain at monomer number separation *n* or contour length separation  = *n*⟨*b*⟩. In order to determine the Kuhn length of our polymer chains, we have simulated melts of *M* = 125 linear chains with *N* = 320 monomers per chain at the same monomer density  (Section 3.2) and for κ_bend_ = 0, 1, 2; then, after equilibration, we have computed numerically eq 18. As shown in Figure 3 (symbols), the chains become increasingly stiffer with κ_bend_ as expected by displaying, in particular, plateau-like regions for large ’s. In analogy to the procedure employed in ref (59), the heights of these plateaus, obtained by best fits to corresponding constants on the common interval  ∈ [150, 200] (Figure 3, dashed lines) provide the values of the corresponding _*K*_’s which are used in this work (see Table 1).

#### Entanglement Length, *L*_*e*_, and Tube Diameter, *d*_*T*_

The entanglement length *L*_*e*_ marks the crossover from entanglement-free to entanglement-dominated effects in polymer melts. In general *L*_*e*_ depends in a nontrivial^57,63^ manner on the microscopic details of the polymer melt, typically the chain Kuhn length _*K*_ and the monomer density ρ. By combining packing arguments^64^ and primitive path analysis,^46^ Uchida et al.^61^ showed that *L*_*e*_ can be expressed as a function of , the number of Kuhn segments in a Kuhn volume at given Kuhn density :
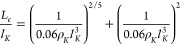
19where the two power laws account for the two limits of *loosely* entangled melts (, exponent = 2) and *tightly* entangled solutions (, exponent = 2/5). By using eq 19, it is a simple exercise to extract *L*_*e*_/_*K*_ and the corresponding number of monomers per entanglement length, *N*_*e*_ ≡ *L*_*e*_/⟨*b*⟩. [Strictly speaking, eq 19 was derived for the entanglement length of a melt of *linear* chains. Yet, recent theoretical work^12,14,23^ on *ring* melts has demonstrated that *the same L*_*e*_ describes the correct contour length where topological constraints affect ring behavior and chains become manifestly crumpled.] We observe (Table 1) that *N*_*e*_ decreases by approximately 1 order of magnitude by the apt fine-tuning of κ_bend_. This means that rings with the same contour length but stiffer will become increasingly entangled.^8^ Finally, we use the definition eq 2:
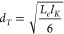
for computing the tube diameters of polymers of different bending stiffnesses. Notice (Table 1) that, by changing chain stiffness, *d*_*T*_ moves from smaller to comparable to _*K*_, meaning that we are effectively able to explore the mentioned crossover from loosely to tightly entangled melts.

#### Entanglement Time, τ_*e*_

The entanglement time τ_*e*_ marks the onset to entanglement-related effects in polymer dynamics. As anticipated in Section 2.1, τ_*e*_ is evaluated numerically as , where *g*_1_(τ) is the monomer time mean-square displacement (eq 8) and *d*_*T*_ is the tube diameter (eq 2). For consistency with the definitions for *L*_*e*_ and *d*_*T*_, notice that *g*_1_(τ) has been calculated on the same dynamic simulations of melts of *linear* chains used for calculating _*K*_.

### 3.5 Computing the Ring Minimal Surface

The minimal surface spanned by a ring polymer was introduced^17,53^ as a quantitative tool for measuring the “exposed” area that each ring offers to its neighbors. In this section, we limit ourselves to summarize the salient numerical aspects of the procedure used to obtain the minimal surface; the interested reader will find a complete overview on minimal surfaces for melts of rings in the mentioned references and also in ref (22).

The search for the minimal surface of a ring polymer is based on a suitable minimization algorithm which works as the following. Essentially, the algorithm is based on successive iterations of triangulations evolving under surface tension by moving the free vertices: each triangle in the initial triangulation is made of two successive monomer positions and the center of the mass of the ring; then it is refined (by subdividing each edge into two edges, creating therefore four triangles out of one) and the surface area minimized by the surface tension flow with restructuring of the mesh. Finally, the algorithm stops when the relative surface area does not change by more than 0.1% over a few tens of additional steps of the minimization procedure. Similarly to the eye of a needle pierced by a thread, the single minimal surface of a given ring can be pierced or *threaded* by the other surrounding polymers in the melt: in particular, once the minimal surfaces of the rings in the melt are determined, it is possible to define in a precise and robust manner what amount of the total contour length of any given ring passes through the minimal surface of another ring.

## 4 Results

### 4.1 Ring Structure

In order to characterize ring structure, we consider the chain mean-square “gyration” radius,
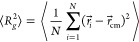
20where  are the monomer coordinates and  is the chain center of mass, and the mean-square “magnetic” radius first introduced in ref (23) and defined as
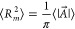
21where, inspired by the analogy to the classical electrodynamics of the magnetic far field generated by a loop carrying a constant electric current,
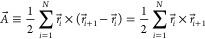
22is the (oriented) area enclosed by the ring. Both quantities are an expression of the average (square) ring size, yet they have different meaning: in particular, eq 21 was introduced to detect and quantify the presence of open loops inside the ring. Numerical values of  and  for melts of rings with different flexibilities are summarized in Table S1 in the SI.

**Figure 4:**
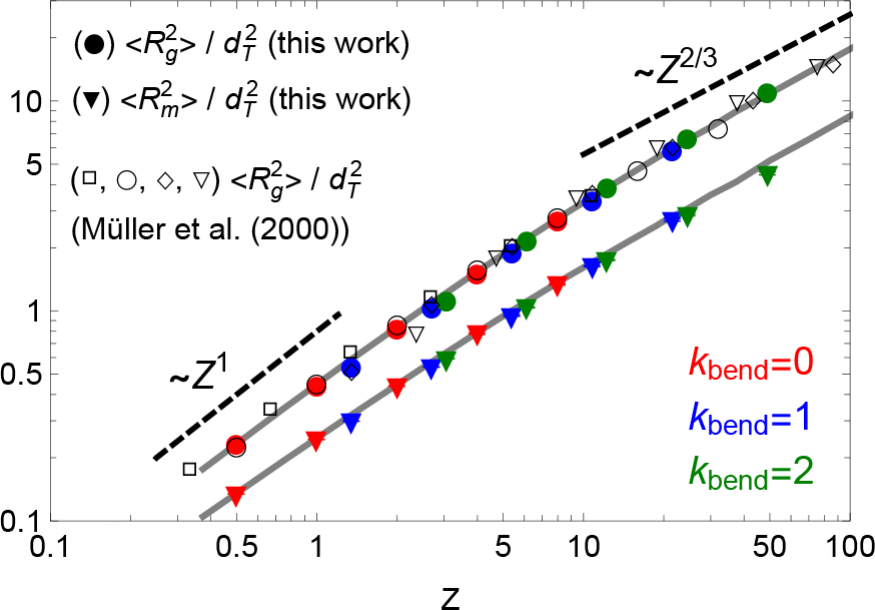
Scaling behavior of the mean-square gyration radius, , and the mean-square magnetic radius, , of ring polymers as a function of the total number of entanglements, *Z* = *L*/*L*_*e*_, of the chain. Error bars are smaller than the symbols size. The empty symbols reproduce the ring data by Müller et al.,^8^ with different symbols for the different Kuhn lengths used in that work (by employing their notation: σ/κ_*B*_*T* = 0 (□), 1 (○), 2 (◇), 3 (▽), from flexible to increasingly stiffer chains). The solid gray lines are for the on-lattice rings on the fcc lattice studied in ref (23).

Figure 4 shows these quantities, rescaled (filled symbols) by the corresponding tube diameters *d*_*T*_ and as a function of the total number of entanglement *Z* = *L*/*L*_*e*_; see Section 2.1 for details and Table 1 for specific values of *d*_*T*_ and *L*_*e*_. For further comparison, we have also included the results for  obtained from Monte Carlo simulations (bond-fluctuation model) of melts of rings by Müller et al.^8^ (open symbols) and the universal curves (solid gray lines) from the “hierarchical crumpling” method by Schram et al.^23^ The excellent matching between these old data sets and the present new data validates our methodology: the average ring size covers the full crossover from Gaussian (∼ *Z*^1^) to compact (∼ *Z*^2/3^) behavior. This is particularly evident for the stiffest rings (κ_bend_ = 2) whose reduced flexibility “helps”, in agreement with Müller et al.,^8^ reaching the asymptotic behavior. The data for the mean-square magnetic radius  rescale equally well and, as noticed in ref (23), with the same large-*Z* behavior . However, a closer look at the ratio  (see Figure S2 in the SI) reveals significant, albeit extremely slow ( with γ ≈ 0.07), power-law corrections which *were not* noticed in the previous study:^23^ instead, a similar result was already described in ref (8), where, however, the authors employed a different definition for the area spanned by a ring which was based on the 2*d* projection of the chain onto a random direction.

**Figure 5:**
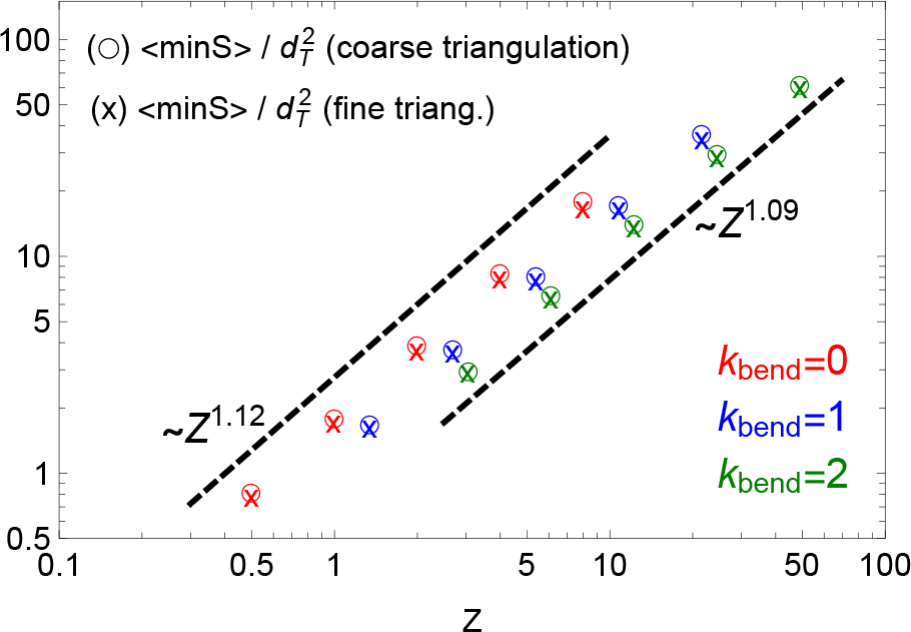
Mean minimal surface area, ⟨min *S*⟩, of ring polymers as a function of the total number of entanglements, *Z*, of the chain. The ○ and × symbols (see legend) are for the two chosen resolutions in the triangulation procedure at the basis of the calculation of the ring minimal surface (see Section 3.5 for details). For the higher resolution, we take two additional refining steps before the minimization procedure stops. Error bars are smaller than the symbols’ size. The power laws (dashed lines) correspond to best fits to the data.

The universal behaviors of the two quantities  and  characterize the chain as a whole, yet, in principle, there could be other length scales below *L*_*e*_ which produce no effect on these quantities but affect and become visible in others. We look then at the average behavior of the ring minimal surface (hereafter, min *S*), a concept introduced^17,22,53^ to quantify the “exposed” area that each ring offers to its neighbors (see Section 3.5 for technical details). Surprisingly, our data (collected in Table S1 in the SI) demonstrate that this is not the case (see Figure 5): in fact ⟨min *S*⟩ ∼ *Z* for the different flexibilities, in agreement with previous results^17^ for off-lattice simulations, but the three data sets—contrary to  and  (Figure 4)—do not collapse onto each other after rescaling in terms of entanglement units *Z* and . Notice that this finding—which looks even more surprising given that both  and ⟨min *S*⟩ are in the end two measures of the ring effective area—is robust and independent of the triangulation resolution employed to calculate the minimal surface of the rings (○ symbols vs × symbols in Figure 5). Note also that ⟨min *S*⟩ is normalized by , which should correct for the different “elementary areas” due to different local structure of the polymer model. [Note also that normalization by the other microscopic length scale, _*K*_, would not imply a collapse either; in fact it would make it even worse as _*K*_ grows with κ_bend_ (see Table 1).]

What could be the physical origin of this discrepancy? In an earlier paper^22^ it was shown that the minimal surface of each ring can be pierced (threaded) a certain number of times by portions of surrounding rings, but that it is necessary to distinguish carefully the contributions from chain contour lengths *z* = /*L*_*e*_ ≲ 1 (*shallow threadings*) from the others. For each ring piercing the minimal surface of another ring, we define^22^ the threading contour length  as the contour length portion of the ring comprised between the two consecutive penetrations *i*-th and *i* + 1-th, which allow us to assign *unambiguously* to either “side” of the other ring’s minimal surface. Based on , we consider the following observables:

1. The mean number of chain neighbors threaded by a single ring, ⟨*n*_*t*_⟩.
2. The mean relative amount of contour length of a ring on one side of another ring’s minimal surface with respect to the other side:

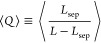
23where the so-called *separation length*,^17,22^
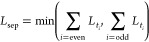
24quantifies the total contour length of one of the two complementary portions (the other has contour length *L* – *L*_sep_) obtained by the passage of the *penetrating* ring through the minimal surface of a *penetrated* ring: i.e., *Q* = 1 simply means that the penetrating ring is half split by the penetrated surface.

3. The mean number of times any ring penetrates the minimal surface of any other ring, ⟨*n*_*p*_⟩.

**Figure 6:**
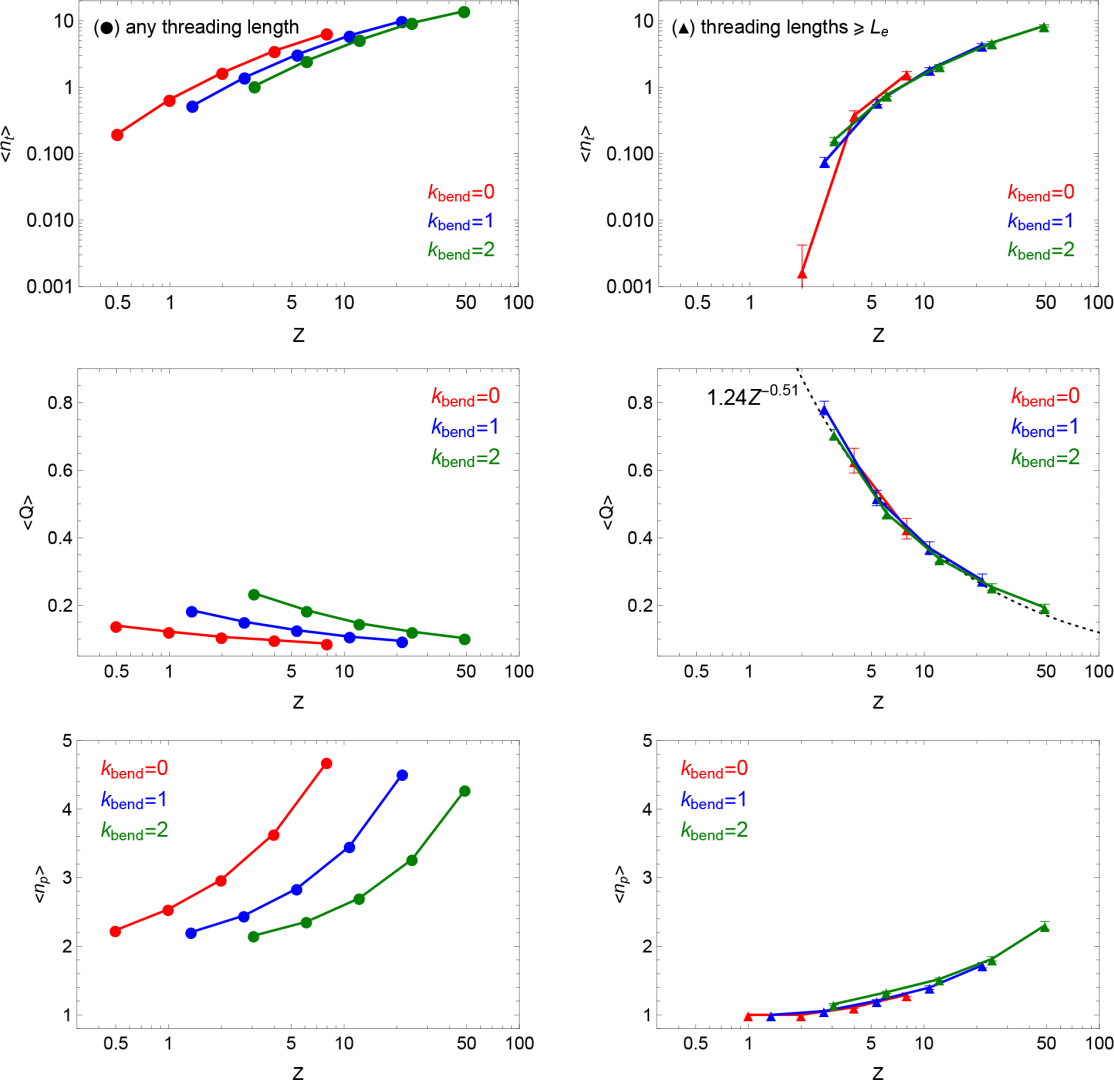
Statistics of threadings by minimal surfaces as a function of the total number of entanglements, *Z*, of each ring in the melt. The data (circles) shown in the left-hand panels (respectively, (up-triangles) in the right-hand panels) have been obtained by including threading lengths *L*_*t*_ of any size (respectively, *L*_*t*_ ≥ *L*_*e*_). Error bars represent the error of the mean calculated from 100 uncorrelated snapshots. (Top) ⟨*n*_*t*_⟩: mean number of rings threaded by a single ring. (Middle) ⟨*Q*⟩: mean relative amount of contour length piercing one ring minimal surface side with respect to the other (eq 23). The dashed (black) line on the right-hand panel corresponds to the best fit to the data. (Bottom) ⟨*n*_*p*_⟩: mean number of times a ring penetrates the minimal surface of any other single ring.

These different quantities may be computed by taking into account all possible threading segments or by excluding the shallow ones, namely, as said, those whose contour lengths are shorter than the entanglement length *L*_*e*_. Noticeably, in the first case there is no evidence for universal collapse (Figure 6, left-hand panels) in any of these quantities. On the contrary (Figure 6, right-hand panels), universal collapse is observed after removing the contribution of the short threading filaments: this points to the fact that the same shallow threadings are responsible for the lack of universality observed for ⟨min *S*⟩ (Figure 5). Assuming that each shallow threading contributes  to the average ⟨min *S*⟩, their removal restores universality almost completely (Figure S3 in the SI). To justify the assumption that each threading does not contribute more than about  to the minimal surface, we measured the mean distance ⟨*d*⟩ between two bonds involved in the threading. We find that indeed ⟨*d*⟩ ≲ *d*_*T*_ (see the values, the corresponding distribution functions, as well as the discussion of the cases with more than two piercings in Figure S4 in the SI), which strongly supports the fact that threadings occur on scales below the entanglement scale. We still find some cases when ⟨*d*⟩ > *d*_*T*_, but these are exponentially rare (see Figure S4 in the SI).

Additionally, restricting the discussion to the universal behaviors, we see that ⟨*Q*⟩ decreases with *Z*, i.e., less material enters the minimal surface of a ring. Yet, the mean number of times, ⟨*n*_*p*_⟩, any ring penetrates the minimal surface of any other single ring increases: notice that these two apparent contradictory features can be easily reconciled by supposing that while retracting from each other (i.e., decreasing ⟨*Q*⟩), rings may at the same time pierce each other more frequently by the increased propensity to form branches at the entanglement scale. Overall, this agrees with the results by two of us (J.S., A.R.)^22^ for off-lattice dynamic simulations of melts of rings. [Notice, however, that in ref (22) we have reported the scaling behavior ⟨*Q*⟩ ∼ *Z*^–0.31^, distinct from the one measured here (⟨*Q*⟩∼ *Z*^–0.51^, see middle right panel in Figure 6). This apparent discrepancy is due to the fact that in ref (22) the mean value ⟨*Q*⟩ includes the contribution of the shallow threadings: in fact, their removal gives identical results to the ones of the present work (data not shown).]

According to the lattice-tree model (Section 2.2), the contour length of each ring above the entanglement length *L*_*e*_ should double-fold along a branched (i.e., tree-like) backbone. We seek now specific evidence of this peculiar organization. As the first of these signatures, we look at the bond-vector correlation function^8,14,23^
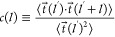
25

**Figure 7:**
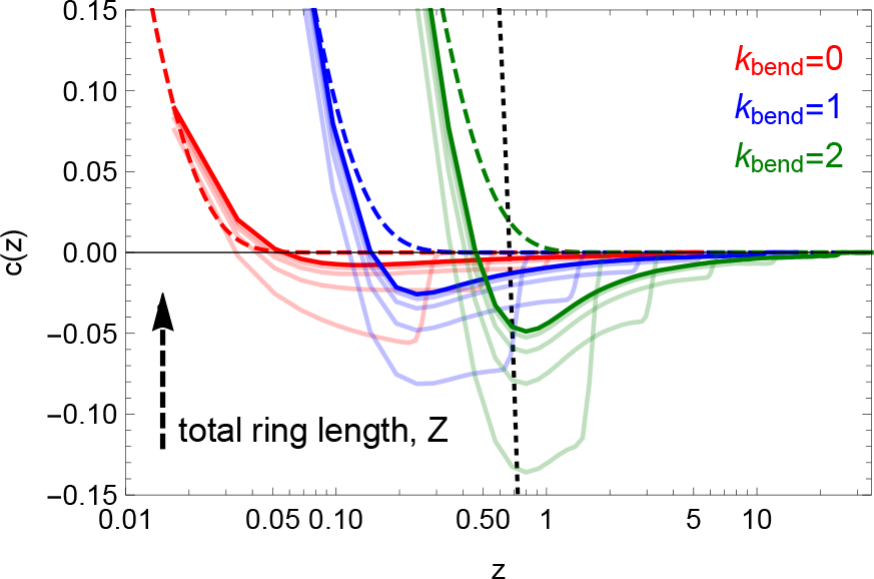
Bond-vector orientation correlation function, *c*(*z*) (eq 25), as a function of the number of entanglements *z* = /*L*_*e*_ along the chain. Lines of equal color are for the same chain stiffness (see legend), full colors are for the longest rings (*N* = 640), while lines in fainter colors are for chains of shorter contour lengths (see arrow’s direction). The long-dashed lines correspond to the exponential decay typical of linear polymers with local stiffness, namely, , where the values for ⟨ cos θ⟩^ring^ are reported in Table 1. The short-dashed line is the analytical function for an exactly double-folded polymer filament of contour length = 2*L*_*e*_ (see the text for details).

Contrary to the known exponentially decaying behavior typical of linear polymers (solid lines vs long-dashed lines in Figure 7) *c*() is manifestly nonmonotonic, showing an anticorrelation well whose minimum becomes more pronounced and locates around *z* = /*L*_*e*_ ≈ 1 with the increasing of the chain stiffness. By using the function  for 0 <  < *L*_*e*_ and  for *L*_*e*_ <  < 2*L*_*e*_ as a toy model for an exactly double-folded polymer filament of contour length = 2*L*_*e*_, it is easy to see that *c*(*z* = /*L*_*e*_) = (1–3/2*z*)/(1 – *z*/2) for 0 < *z* < 1 and *c*(*z*) = −1 for 1 ≤ *z* < 2, i*.*e*.*, *c*() displays an anticorrelation minimum at *z* = 1 or  = *L*_*e*_ (short-dashed line in Figure 7). A deep anticorrelation well is particularly visible in short rings (at a given κ_bend_), while in larger rings the effect is smoothed, arguably because thermal fluctuations wash out such strong anticorrelations.

As a second signature, we look at the mean contact probability,^52^ ⟨*p*_*c*_()⟩, between two monomers at given contour length separation  defined as
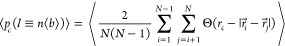
26where Θ(*x*) is the usual Heaviside step function and the “contact distance” *r*_*c*_ between two monomers is taken equal to one lattice unit, *a*.

**Figure 8:**
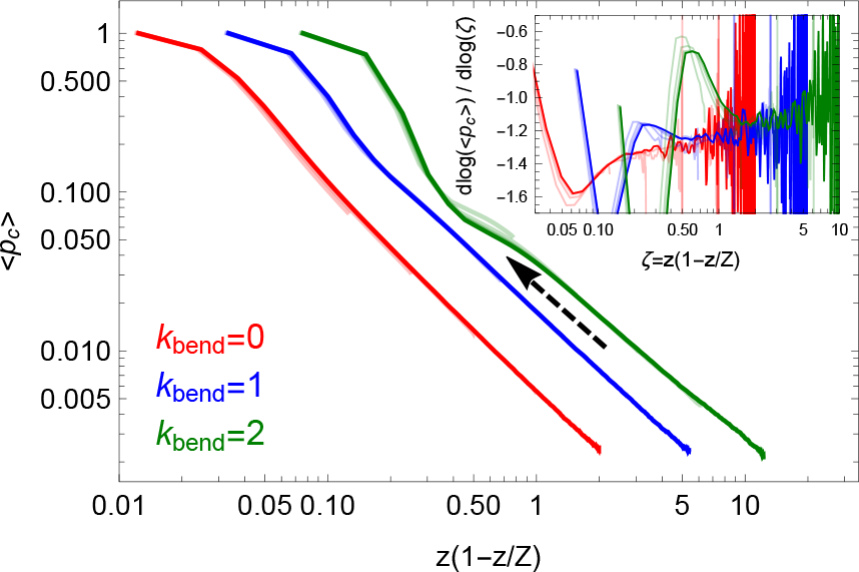
Mean contact probabilities, ⟨*p*_*c*_()⟩ (see eq 26), as a function of the “effective” ring contour length ζ ≡ *z*(1 – *z*/*Z*). The arrow points at the leveling of the curves around *z* ≈ 1. Inset: local differential exponent . Color code is as in Figure 7.

Reducing finite ring size effects by plotting ⟨*p*_*c*_⟩ in terms of the variable^24^ ζ ≡ *z*(1 – *z*/*Z*), the data from the different rings (see Figure S5 in the SI) form three distinct curves according to their Kuhn length (Figure 8). Notably these curves display the asymptotic power-law behavior ∼ζ^–γ^ with scaling exponent γ close to 1, as reported in the past.^9,14,35^ At the same time, the stiffer chains with κ_bend_ = 2 display a short, yet quite evident, leveling of the contact probability curves around *z* ≈ 1 (see arrow’s direction), which is clearly compatible with double-folding on the entanglement scale.

**Figure 9:**
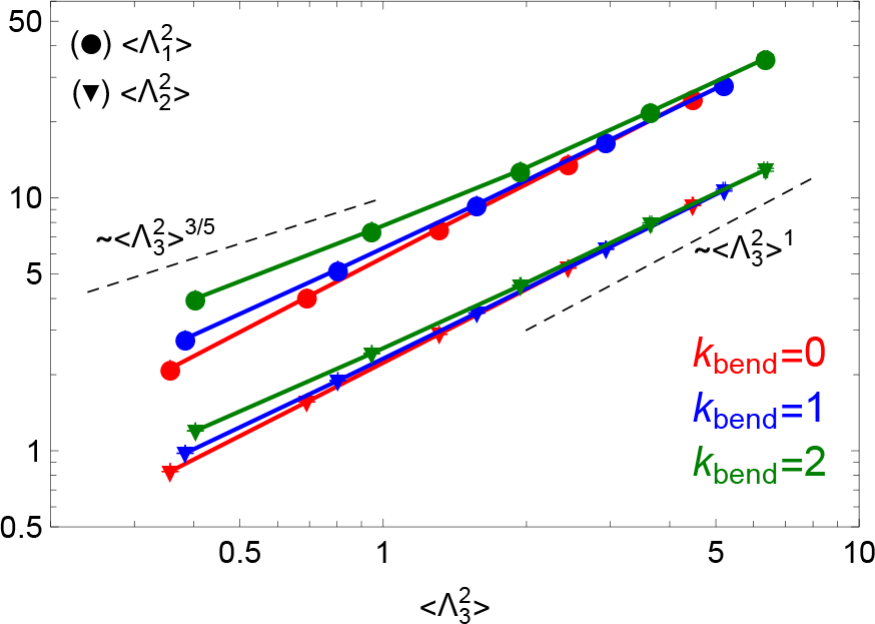
Mean eigenvalues,  and , of the gyration tensor eq 27 as a function of the smallest eigenvalue . The dashed lines are the predicted limits of small and large length scales.

However, we show now that the most compelling evidence for double-folding comes from examining the average polymer *shape*. To this purpose, we introduce the 3 × 3 symmetric gyration tensor  whose elements are defined by
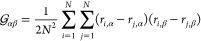
27where *r*_*i*,α_ with α = *x*, *y*, *z* are the Cartesian components of the spatial position of monomer *i*: the trace of the tensor , , is equal to , and the three ordered eigenvalues, , quantify the spatial variations of the polymer along the corresponding principal axes, i.e., the instantaneous shape of the chain. Polymers are ellipsoidal on average,^51^ with mean values  (see values in Table S1 in the SI): similarly to  (Figure 4), we do expect a scaling curve for each  in universal units *Z* and *d*_*T*_ and, for *Z* ≫ 1, . In general our data (see Figure S6 in the SI) reflect well this trend, except for the smallest mean eigenvalue , which displays systematic deviations from the asymptotic behavior which persist for chain sizes well above the entanglement threshold *Z* ≈ 1. Interestingly, these deviations are *quantitatively* consistent with the scaling properties of rings being double-folded on an underlying tree-like structure (Section 2.2): in fact, according to Figure S6 in the SI and for the given mean path length ⟨*L*_tree_⟩ ∼ *Z*^ρ^ (eq 6) of the “supporting” tree, we can write the expressions
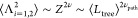
28
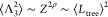
29where the latter is equivalent to assuming local stiff behavior at small contour length separations. Equations 28 and 29 are just equivalent to
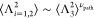
30with ν_path_ = 3/5 (eq 7). Our data are well described by eq 30 (see Figure 9) before the crossover to the asymptotic regime, , finally takes place. Notice that the value of ν_path_ agrees well with that of the annealed tree model.^13^ Note also that here we observe the exponent clearly only for the stiffest system. This might explain why the work^19^ that analyzed only simulations^9^ with low stiffness (roughly, between our κ_bend_ = 0 and κ_bend_ = 1 in terms of *L*_*e*_) reports that they do not see ν_path_ (there measured as the scaling of the chain *primitive path* with the contour length).

In conclusion, the *universal* scaling features of the static quantities and the bond correlations reveal double-folded tree-like structure on scales above *L*_*e*_, with indications  that the trees are of the annealed tree type.^13^ Conversely, the *nonuniversal* features (⟨min *S*⟩) and threading analysis show that the trees are threaded on scales smaller than the tube diameter *d*_*T*_, hence reconciling the tree picture with that of the threaded conformations. At the same time the “depth” of threadings can be (Figure 6) longer than *L*_*e*_, giving hints on its relevance for the dynamic features. Therefore, in the next section we explore the consequences of the double-folding and threadings on the dynamic properties of the melts.

### 4.2 Ring Dynamics

**Figure 10:**
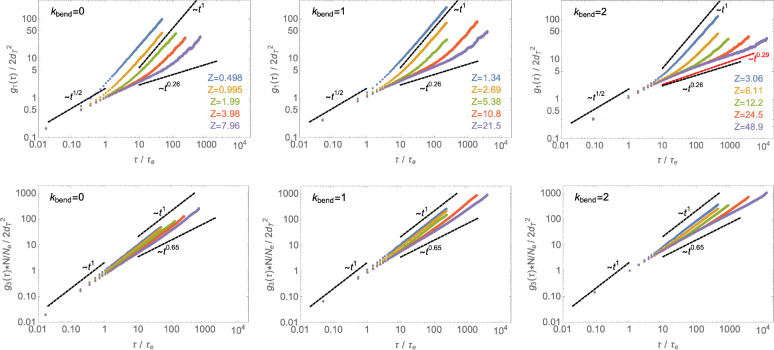
Monomer time mean-square displacement, *g*_1_(τ) (eq 8), and chain center of mass time mean-square displacement, *g*_3_(τ) (eq 9). Symbols of the same color are for the same number of monomers *N*, and the corresponding number of entanglements *Z* per chain is indicated in the legends. The dashed black lines correspond to the dynamic predictions of the lattice tree model, eqs 10 and 11 with ν = 1/3 (eq 5) and ρ = 5/9 (eq 6). The dashed red line in the top-right panel corresponds to the theoretical prediction of the FLG model^19^ by Ge et al.

As briefly discussed in Section 2.3, the lattice-tree model predicts universal dynamic chain behavior for length scales ≳ *d*_*T*_ and time scales ≳ τ_*e*_. Recent numerical works employing off-lattice molecular dynamics simulations^10^ and lattice models^27^ agree well with these predictions. In order to compare the dynamic behavior of the present systems to the results of the lattice-tree model (namely, eqs 10 and 11) with ν = 1/3 (eq 5) and ρ = 5/9 (eq 6), we look then at the monomer mean-square displacement, *g*_1_(τ), and the center of mass mean-square displacement, *g*_3_(τ), and plot them in properly rescaled length and time units by using the values of the parameters *d*_*T*_ and τ_*e*_ reported in Section 3.4. Figure 10 demonstrates the correctness of the rescaling procedure and, in line with previous works,^10,27^ the good agreement between our simulations (symbols) and the lattice-tree predictions (dashed black lines), in particular for melts of rings with κ_bend_ = 2 and *Z* ≈ 50. Notice though that our data also match well the predictions of the FLG model^19^ by Ge et al. (dashed red line in the top-right panel), since the two scaling exponents (0.29 vs 0.26, respectively) are within 10% of each other and, hence, beyond the present accuracy of our data.

We conclude now the paper by investigating the implications of threading and double-folding for ring dynamics. While slightly off-topic with respect to the main body of this work, we think nonetheless that the results we are going to discuss represent a valid complement to it.

The formation of topological links via threadings has been held responsible of the unique rheological properties of melts of rings as, for instance, the unusually strong extension-rate thickening of the viscosity in extensional flows.^26^ Notably, it was conjectured^36^ that inter-ring threadings should form a network of topological obstacles which, by percolating through the entire melt, should sensibly slow down the relaxation of the system, not dissimilar from what happens in those materials undergoing the glass transition. While direct proof of this *topological glass* is currently missing, recently Michieletto and others^37,38^ gave indirect evidence of this through the following numerical experiment: they froze (pinned) a certain fraction *f*_*p*_ of rings in the melt and reported that the dynamics of the remaining ones is considerably reduced if not frozen at all. In corresponding melts of linear chains this effect is not seen, so they attributed the observed slowing down to the presence of threadings present in rings but absent in linear chains.

**Figure 11:**
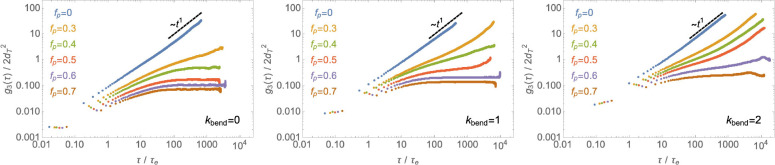
Effects of random pinning on chain dynamics. Chain center of mass time mean-square displacement, *g*_3_(τ) (eq 9), at different pinning fractions, *f*_*p*_, and chain flexibilities, κ_bend_ (see legends). Results correspond to melts with the same number of entanglements, *Z* ≈ 8, per individual chain.

**Figure 12:**
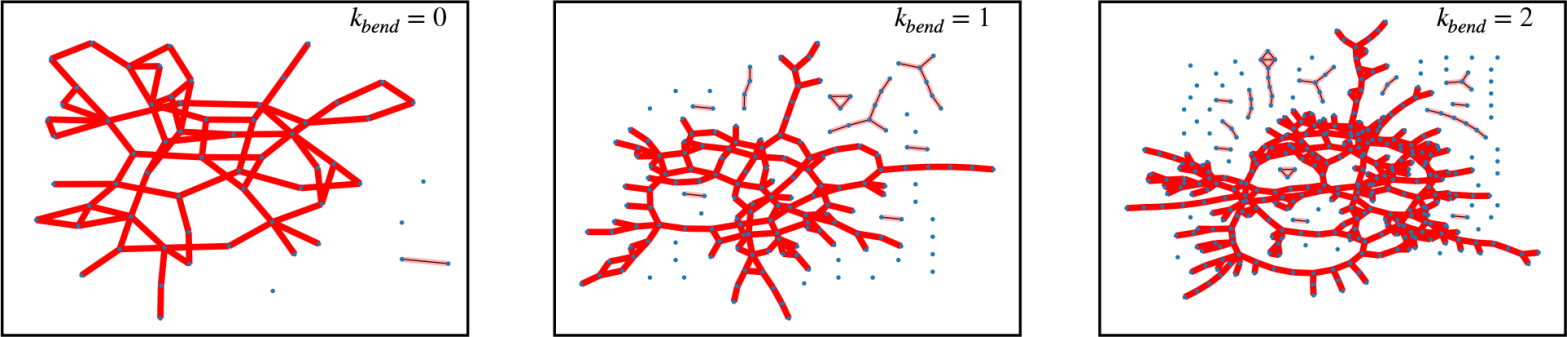
Network representation of ring melts with the same number of entanglements, *Z* ≈ 8, per individual chain and for different chain flexibilities κ_bend_ (see legends). Each node of the network represents a given ring of the melt, and the bond between two nodes means that one of the two ring is threading the (minimal surface of the) other (see the discussion in Section 4.1). For constructing the networks, we have included threading lengths *L*_*t*_ > *L*_*e*_ for which universal scaling behavior emerges (see Section 4.1).

In the numerical experiments discussed here, while double-folding is “enhanced” (Section 4.1) by the chain local stiffness, long threadings, with *L*_*t*_ > *L*_*e*_, which should be the ones affecting the dynamics of the system, are universal. At this level, it is unclear what the impact of these features is on the dynamics and whether or not they can be distinguished. To get some insight into this question, we have taken inspiration from the mentioned pinning numerical experiments^37,38^ and performed additional simulations of melts of rings for κ_bend_ = 0, 1, 2 with the same number of entanglements *Z* = 8 (to ensure the same large-scale behavior, see Figure 4) and for ring pinning fractions *f*_*p*_ from 0 (i.e., no pinning) to 70%. Then we monitored the mean-square displacement, *g*_3_(τ), of the center of mass of the nonpinned rings. As shown in Figure 11, our results are consistent with the proposed picture: more flexible rings (κ_bend_ = 0, left panel) which we have interpreted as less double-folded are already completely frozen at *f*_*p*_ ≃ 30–40%, while the stiffer (and comparably more double-folded) rings (κ_bend_ = 2, right panel) require *f*_*p*_ ≃ 60–70%. Importantly, these specific fractions do not seem to depend on the finite size of our systems: dynamic runs with smaller (and, arguably, more finite-size dependent) systems give similar values for the freezing fractions (see Figure S7 in the SI). Finally, we see that this is compatible with a network of percolating topological constraints. To this purpose, we represent the melts as networks where each node corresponds to a ring, and we draw a link between two nodes (i.e., rings) whenever the minimal surface of one of the two rings is pierced by a threading segment of the other ring of contour length *L*_*t*_ > *L*_*e*_ (i.e., we neglect the shallow threadings). A picture of these networks is shown in Figure 12: it is seen that, at increasing chain stiffness, the fraction of rings globally connected through threadings sensibly decreases with chain stiffness, from  for κ_bend_ = 0 to  for κ_bend_ = 1 and κ_bend_ = 2; that is, the network of threadings is percolating less through the stiffer melts. [In principle, these percentages may be affected by finite-size effects due to the limited number of chains of our melts. Nonetheless, our conclusions should be fairly robust.] Notice that this decrease corresponds roughly to the fractional amount  of rings which need to be additionally pinned in the stiffer systems compared to κ_bend_ = 0 in order to get complete freezing (Figure 11). To summarize, flexible melts showing higher propensity to shallow threadings (Figure 6) are more strongly affected by the pinning, pointing to a possible dynamical role of the shallow threadings. In contrast—yet consistent with the pinning fraction—the deep threadings in flexible melts form more connected networks in comparison to the stiffer melts, seemingly suggesting that these alone are the relevant ones in the pinning experiment. The latter result is however surprising, because the deep threadings were found to behave universally for the systems with different stiffness (see Figure 6). We leave this puzzle for future work.

Another view on the results of our pinning experiments shows that the threading constraints are a relevant physical feature that needs to be taken into account to refine models for the ring dynamics. As we mentioned (eq 14), the ring relaxation time with (θ = 1) or without (θ = 0) the tube dilation can be written^19^ as
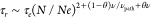


Notably, the pinning of a large fraction *f*_*p*_ of rings must stop the dilation, because the pinned chains around a mobile ring cannot move, hence impose constraints at all times. The plateau of the *g*_3_ that we observe indicates a divergence of the relaxation time, but the FLG as well as the lattice models give a finite prediction for the relaxation time even for inhibited dilation θ = 0. Indeed this is the consequence of neglecting of the threadings. Although the FLG model is built on the full tube dilation in the melt, and therefore, its comparison to the pinning with a limited dilation is problematic, its general formulation (eq 14) allows considering a partial (θ < 1) or no tube dilation. Last but not the least, even though both the lattice-tree and the FLG model underestimate the scaling exponent of the diffusion coefficient with *N* in comparison to simulations and experiment, a similar underestimation of the scaling exponent of the diffusion coefficient is found in *linear* melts when compared to a naïve reptation theory.^2^ The agreement between the theory and the experiment is restored when contour length fluctuations and tube dilation are incorporated. In the rings, the FLG model already includes the tube dilation, yet the prediction of the exponent does not agree with the experiment, which indicates that this is due to the neglect of the threadings. Another explanation of the discrepancy might be the correlation hole effect as indicated in ref (33). Whether this is independent of threadings remains to be elucidated.

## 5 Discussion and Conclusions

The conformational properties of unknotted and non-concatenated ring polymers in dense melts represent one of the remaining unsolved challenges in polymer physics. In this work, in particular, we have focused on which properties of the rings can be interpreted as signatures of the hypothesis proposed long ago^4,5,7^ that the polymer fiber double-folds around a branched (lattice-tree) path.

In this respect, global observables like the polymer mean-square gyration radius or the mean-square magnetic radius ( and , Figure 4) are very useful for model validation but otherwise offer little insight.

On the contrary, robust evidence for double-folding comes by exploiting the mean polymer shape (Figure 9). The three principal axes of the polymer are very different in size and, for relatively moderate polymer lengths, they are not at the same “point” of the crossover to asymptotic behavior: in particular, on the studied range where the largest and the smallest axes are not proportional to each other, we have shown that their functional relation is in *quantitative* agreement with the lattice-tree model. This argument is proposed here for the first time, and it could be useful to revise data relative to other polymer models at its light.

Additional signatures of double-folding on polymer contour lengths *z* = /*L*_*e*_ ≲ 1 become manifested also in the characteristic negative well of the bond-vector orientation correlation function (Figure 7) and in the “softer” slope of the mean contact probability ⟨*p*_*c*_()⟩ (Figure 8) displayed before the asymptotic ∼ ^–1^ power-law behavior effectively takes place. Intriguingly, the reported ⟨*p*_*c*_()⟩ for chromatin fibers measured in conformation capture experiments^52^ displays the same systematic two-slope crossover^65^ for  ≲ 10^5^ base pairs, i.e., below the estimated^34^ entanglement length of the chromatin fiber. Notice that, in this respect, the formulated hypothesis that such a “shoulder” in the contact probability derives from active loop-extrusion^65^ provides a dynamic explanation about how (double-folded) loops can form; otherwise it is not needed *per se* in order to explain the observed trend in the contact probabilities.

Overall it is worth stressing that these features, that we interpret as manifestations of double-folding, can be made explicit only by introducing some (even if only moderate) bending penalty to the polymer elasticity.

Using minimal surfaces we confirm (Figure 5) that rings may reduce their threadable surfaces via double-folding on the entanglement scale and that the piercings of the minimal surface occur only on the scale below the entanglement tube radius. Yet, the threading loops can be numerous and their length can exceed the entanglement length, but these threading features (Figure 6 and Figure S3) evaluated on length scales larger than one entanglement length behave universally. The universal behavior shows the applicability and the relevance^47^ of the (linear) entanglement scale to ring polymer melts.

From the point of view of ring dynamics, we have reported that single monomer and global chain motions are in good agreement (Figure 10) with the theory stating that the dominant mode of relaxation is mass transport along the mean path of the underlying tree. Yet the threading constraints can cause divergence of the relaxation time if a fraction of rings is immobilized, a feature not predicted by the theories. Revisiting the theories to incorporate threading constraints explicitly might also help to clarify if the conjecture on the existence of topological glass in equilibrium is valid.

## Supporting Information Available

Table of mean values of structural properties of ring polymers, monomer mean-square displacement *g*_2_ in the frame of the ring center of mass, ratios of mean-square gyration radius and mean-square magnetic radius, mean minimal surface after removing the contribution of shallow threadings, distribution functions of distances *d* between subsequent piercings, mean contact probabilities, mean-square eigenvalues of the gyration tensor, effect of random pinning on smaller melts (PDF)
