# Supplementary material for: Entanglement Length Scale Separates Threading from Branching of Unknotted and Non-concatenated Ring Polymers in Melts
Source: Macromolecules. 2022 Nov 28;55(23):10723–36. doi: 10.1021/acs.macromol.2c01264 (PMC9753756; doi:10.1021/acs.macromol.2c01264)
Supplement: Supplementary file 1 — ma2c01264_si_001.pdf [file ma2c01264_si_001.pdf]

Entanglement length scale separates threading from branching of unknotted and non-concatenated ring polymers in melts – Supporting Information –

Mattia Alberto Ubertini, Jan Smrek, Angelo Rosa*

N	M	$\langle R_g^2 \rangle$	$\langle R_m^2 \rangle$	$\langle$minS$\rangle$	$\langle \Lambda_1^2 \rangle$	$\langle \Lambda_2^2 \rangle$	$\langle \Lambda_3^2 \rangle$
				$\kappa_{\text{bend}} = 0$			
40	1000	3.3007 ± 0.0007	1.8937 ± 0.0008	11.60 ± 0.05	2.1160 ± 0.0007	0.8280 ± 0.0003	0.35669 ± 0.00008
80	500	6.304 ± 0.004	3.447 ± 0.004	25.6 ± 0.2	4.052 ± 0.004	1.560 ± 0.001	0.6912 ± 0.0003
160	250	11.76 ± 0.02	6.19 ± 0.02	55.6 ± 0.4	7.56 ± 0.02	2.891 ± 0.005	1.310 ± 0.001
320	125	21.48 ± 0.08	11.0 ± 0.1	119.7 ± 1.2	13.74 ± 0.07	5.29 ± 0.02	2.447 ± 0.005
640	62	38.6 ± 0.2	18.9 ± 0.2	256.2 ± 2.8	24.7 ± 0.2	9.37 ± 0.05	4.47 ± 0.01
				$\kappa_{\text{bend}} = 1$			
40	1000	4.133 ± 0.001	2.262 ± 0.002	12.82 ± 0.07	2.772 ± 0.001	0.9778 ± 0.0004	0.38363 ± 0.00008
80	500	7.915 ± 0.008	4.09 ± 0.01	28.6 ± 0.2	5.220 ± 0.006	1.893 ± 0.002	0.8028 ± 0.0004
160	250	14.52 ± 0.02	7.19 ± 0.05	61.9 ± 0.6	9.43 ± 0.02	3.506 ± 0.007	1.573 ± 0.002
234	171	19.97 ± 0.06	9.70 ± 0.04	94.3 ± 0.8	12.95 ± 0.03	4.834 ± 0.008	2.223 ± 0.002
320	125	25.8 ± 0.1	12.38 ± 0.07	132.3 ± 1.4	16.67 ± 0.08	6.28 ± 0.02	2.936 ± 0.006
640	62	44.3 ± 0.5	20.5 ± 0.3	279.7 ± 3.3	28.1 ± 0.4	10.73 ± 0.09	5.20 ± 0.03
				$\kappa_{\text{bend}} = 2$			
40	1000	5.600 ± 0.006	2.935 ± 0.005	14.8 ± 0.1	3.998 ± 0.004	1.199 ± 0.002	0.4030 ± 0.0003
80	500	10.82 ± 0.02	5.15 ± 0.03	33.0 ± 0.3	7.45 ± 0.02	2.426 ± 0.007	0.946 ± 0.002
104	385	13.56 ± 0.02	6.27 ± 0.02	44.1 ± 0.4	9.21 ± 0.01	3.092 ± 0.004	1.261 ± 0.001
160	250	19.4 ± 0.2	8.66 ± 0.06	70.7 ± 0.7	12.87 ± 0.07	4.50 ± 0.02	1.943 ± 0.006
320	125	33.2 ± 0.3	14.2 ± 0.1	148.7 ± 1.8	22.0 ± 0.3	7.90 ± 0.07	3.65 ± 0.02
640	62	54.9 ± 1.4	22.4 ± 0.4	308.8 ± 3.5	35.6 ± 1.3	12.9 ± 0.2	6.36 ± 0.06

TABLE S1. Conformational properties of melts of ring polymers with bending stiffness κ_{bend}. N: number of monomers per chain; M: number of chains in the melt; $\langle R_g^2 \rangle$: mean-square gyration radius (Eq. (19) in the main paper); $\langle R_m^2 \rangle$: mean-square magnetic radius (Eq. (20) in the main paper); $\langle$minS$\rangle$: mean minimal surface; $\langle \Lambda_\alpha^2 \rangle$: mean value of the α-th eigenvalue ($\alpha = 1, 2, 3$) of the gyration tensor $\mathcal{G}_{\alpha\beta}$ (Eq. (26) in the main paper); The reported values with the corresponding error bars have been rounded to the first decimal digit. The data for $\langle$minS$\rangle$ are for a coarse resolution of the triangulation procedure adopted to calculate the minimal surface of a ring polymer (see Ref. [1] for the technical details). A finer triangulation gives essentially the same values ("$\circ$-symbols" vs. "$\times$-symbols" in Fig. 5 in the main paper) which we have not reported explicitly in this Table.

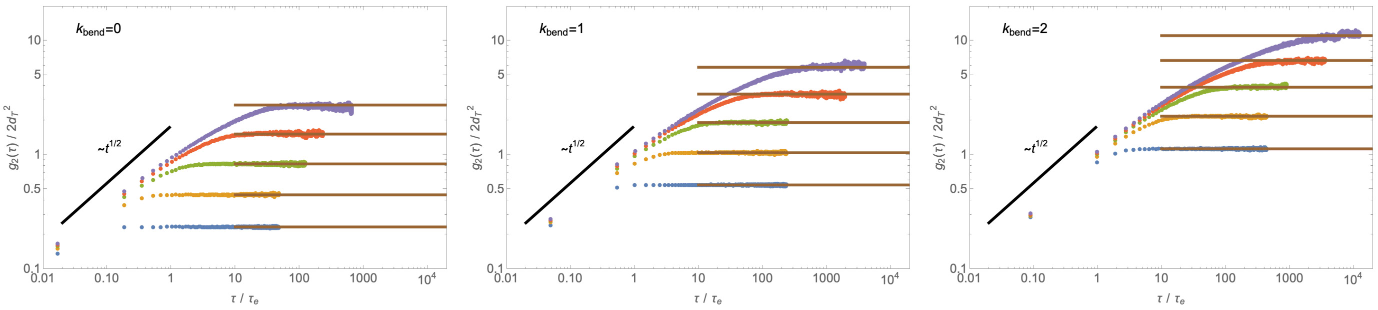

FIG. S1. Monomer time mean-square displacement, $g_2(\tau)$ (see Eq. (12) in the main paper), in the frame of the centre of mass of the corresponding chain. The horizontal lines are for the stationary values, $= 2\langle R_g^2(N)\rangle$ (see Sec. 2.3 in the main paper), reported in Table S1. The dashed black lines correspond to the initial Rouse behavior where $g_2(\tau) \sim g_1(\tau)$ (see Eq. (10) in the main paper). Color code is as in Fig. 10 in the main paper.

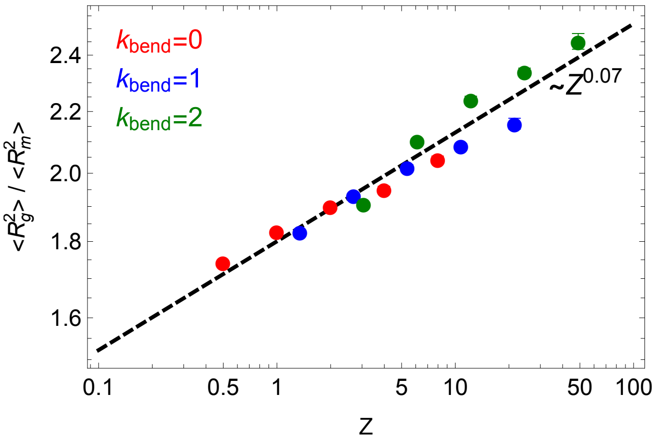

FIG. S2. $\langle R_g^2 \rangle / \langle R_m^2 \rangle$: ratio of the mean-square gyration radius (Eq. (19) in the main paper) to the mean-square magnetic radius (Eq. (20) in the main paper) as a function of the total number of entanglements, $Z = L/L_e$, of the chain. Notice the *weak* increasing of the ratio with Z (the straight line is obtained by best fit of the data to a simple power-law).

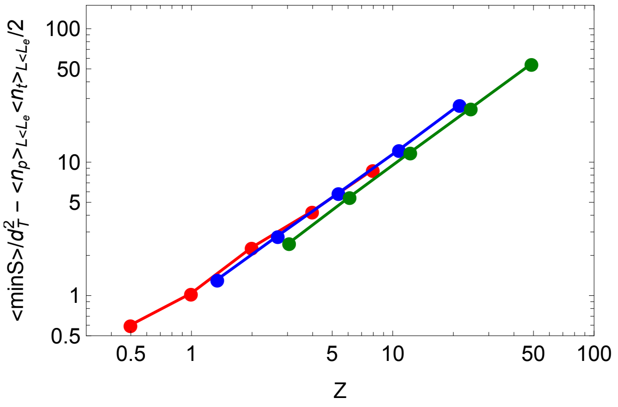

FIG. S3. Mean minimal surface area, $\langle \mathrm{minS} \rangle$, of ring polymers as a function of the total number of entanglements, Z, of the chain after subtracting the "contribution" to the area by short (or, *shallow*) threadings with contour length $L < L_e$. This contribution is calculated by assuming that each threading span an area $\sim d_T^2$ and there are $\sim \langle n_p \rangle_{L<L_e} \langle n_t \rangle_{L<L_e}/2$ of such threadings spanning the minimal surface of each ring. $\langle n_t \rangle_{L<L_e}$ and $\langle n_p \rangle_{L<L_e}$ are (as in Fig. 6 in the main text, but here restricted to shallow threadings) the mean number of rings threaded by a single ring (which equals the mean number of threadings received by a single ring) and the mean number of times a ring penetrates the minimal surface of any other ring, while the "1/2"-factor takes into account the fact that a single threading loops counts as twice in terms of contacts on the minimal surface.

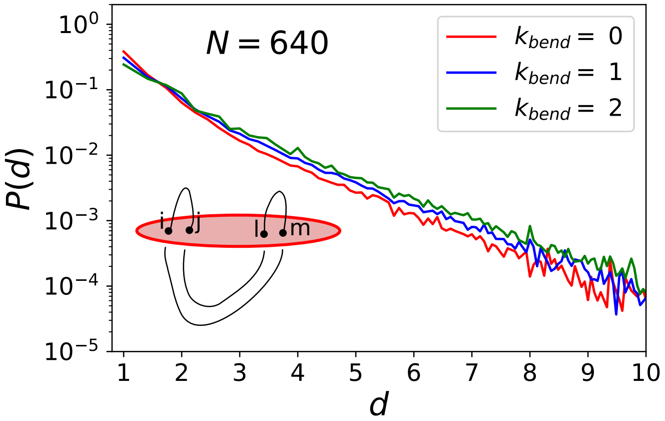

FIG. S4. Distribution functions of distances d between subsequent piercings on the minimal surfaces of rings with $N = 640$ and different flexibilities. Clearly the distributions are exponential. The mean values are $\langle d \rangle = 1.87$, 2.1 and 2.33 (in units of lattice site a) for $k_{\rm bend} = 0$, $k_{\rm bend} = 1$ and $k_{\rm bend} = 2$ respectively. Interestingly, in contrast to the tube diameter d_T, $\langle d \rangle$ grows with the stiffness. Note that, in the case of more than two piercings per surface, there are two different sets of such distances depending on the parity of the piercings (see the inset). For example (see the sketch in the inset) if four bonds i,j,l,m are piercing, one can consider the distances $d(i,j)$ (*i.e.*, the distance between bonds i and j) and $d(l,m)$, or alternatively, $d(j,l)$ and $d(i,m)$. In such cases, we take into account the set with smaller mean ($d(i,j)$ and $d(l,m)$ for the case in the inset). The reason behind this choice is that we want to use $\langle d \rangle^2/2$ as a proxy for the area per piercing. Hence if pairs of piercings are distant from each other (as is the case of (i,j) being far from (l,m)), they represent two uncorrelated events ((i,j) being uncorrelated to (l,m)) and the square of the distance between them (*e.g.*, $d(j,m)$) does not represent the increase of the minimal surface.

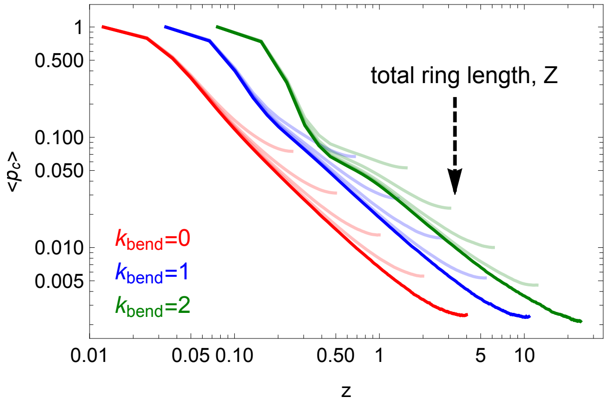

FIG. S5. Mean contact probabilities, $\langle p_c(\ell)\rangle$, as a function of the contour length separation in number of entanglements, $z \equiv \ell/L_e$. Lines in full colors are for the longest rings ($N = 640$) while lines in fainter colors are for chains of shorter contour lengths (see arrow's direction).

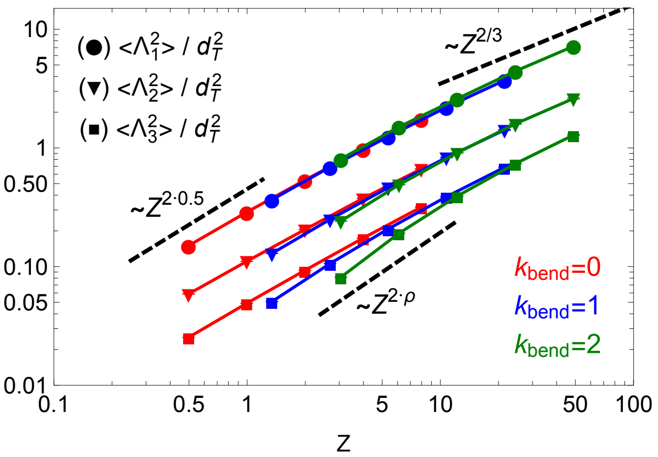

FIG. S6. Scaling behavior of the mean-square eigenvalues $\langle \Lambda^2_{\alpha=1,2,3} \rangle$ of the gyration tensor (Eq. (26) in the main paper) of untangled ring polymers as a function of the total number of entanglements, $Z = L/L_e$, of the chain. Notice the large finite-size corrections to scaling for the smallest eigenvalue $\langle \Lambda_3^2 \rangle$ and its behavior $\sim Z^{2\rho} \sim \langle L_{\rm tree} \rangle^2$ (Eqs. (6) and (28) in the main paper) for small to moderate number of entanglements per chain Z. Error bars are smaller than the symbols size.

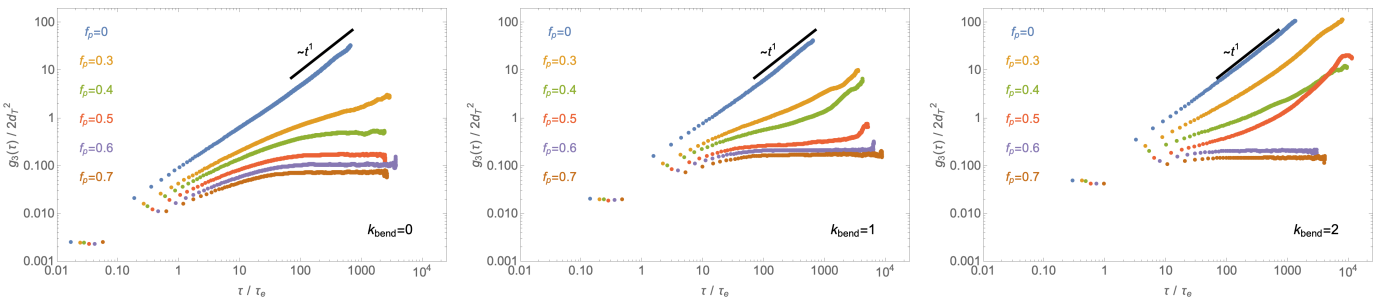

FIG. S7. Effects of random pinning on chain dynamics. Same as in Fig. 11 in the main paper, but for melts containing fewer chains (for implementation details, see Sec. 3.2 in the main paper).

[1] J. Smrek and A. Y. Grosberg, ACS Macro Lett. **5**, 750 (2016).
